# Optimization of a Canine-Specific Ceramide Complex and Evaluation of Its Effects on In Vitro and In Vivo Canine Skin

**DOI:** 10.3390/ani16142148

**Published:** 2026-07-10

**Authors:** Hee Yeon Cho, Bo-Eun Kim, Eunjin Park, Minji Kim, Younhwa Nho, Hyunna Kim, Seunghyun Kang, Chunho Park, Kyung Eun Lee

**Affiliations:** 1 R&I Center, COSMAX BTI, Seongnam 13486, Republic of Korea; heeyeon.cho@cosmax.com (H.Y.C.);; 2 R&I Center, COSMAX, Seongnam 13486, Republic of Korea

**Keywords:** ceramide, dog skin, atopic dermatitis, skin barrier

## Abstract

Alterations in the ceramide (CER) profile have been associated with atopic dermatitis in dogs; however, the specific effects of CER subclasses on canine keratinocytes remain unclear. This study aimed to optimize a CER complex and evaluate its effects on cytokine expression, CER synthesis, and skin barrier formation using canine epidermal keratinocyte progenitor (CPEK) cells and a reconstructed canine epidermis (RCE) model. Among various CER combinations tested, only the mixture of CER [NS] and [EOP] significantly reduced proinflammatory cytokine mRNA levels in *Staphylococcus pseudintermedius*–stimulated CPEK cells. Further optimization revealed that a 4:1 ratio of CER [NS] to [EOP] (cCER complex) markedly decreased CCL17 and CCL22 mRNA expression while upregulating keratin 10, ceramide synthase 3 (CERS3), and ceramide synthase 4, as well as enzymes involved in both de novo and salvage CER biosynthesis pathways. In the RCE model, cCER administration improved stratum corneum organization and increased the expression of loricrin, CERS3, and CER. In vivo application of cCER in dogs accelerated skin barrier repair and improved hair condition. These findings suggest that a CER [NS]/[EOP] mixture represents a promising therapeutic option with anti-inflammatory and skin barrier-enhancing effects for canine atopic dermatitis.

## Simple Summary

Atopic dermatitis is a common and chronic skin disease in dogs, characterized by persistent itching, skin inflammation, and a weakened skin barrier. Ceramides are fatty molecules that naturally occur in the skin and play an essential role in maintaining the skin’s protective barrier. In dogs with atopic dermatitis, ceramide composition is often abnormal, which may worsen skin barrier dysfunction. This study aimed to identify the most effective combination of ceramide types to reduce skin inflammation and restore the skin barrier in dogs. Using dog skin cells grown in the laboratory and a laboratory-based dog skin model, various ceramide mixtures were evaluated. A specific 4-to-1 mixture of two ceramide types was found to significantly reduce inflammatory signals in skin cells exposed to a common skin bacterium, while also promoting the production of proteins essential for a healthy skin barrier. When this ceramide mixture was applied to dogs for four days, it accelerated skin barrier recovery and improved hair quality. These results suggest that this optimized ceramide combination may offer an effective approach to managing skin inflammation and barrier dysfunction in dogs with atopic dermatitis.

## 1. Introduction

Ceramides (CER) play a crucial role in maintaining skin barrier function by preventing transepidermal water loss (TEWL) and protecting against the invasion of external pathogens and allergens [1]. CERs account for approximately 50% of intercellular lipids and form the lipid layer of the stratum corneum (SC) [2]. Previous studies have reported a significant decrease in CER subclasses in the skin of dogs with skin disorders such as atopic dermatitis (AD) and seborrhea [3,4,5].

Because multiple factors contribute to the pathogenesis of canine inflammatory skin diseases, including AD, a multidisciplinary therapeutic approach is required [6]. Therefore, the development of adjunctive therapies to support the effects of other medications has increased in recent years. As an adjunctive therapy, CERs are frequently used to enhance skin hydration and barrier function. However, despite the widespread application of CERs in skincare products for dogs, most commercial formulations containing CER variants are optimized for human skin, particularly CER [NP] and CER [AP]. Previous research indicates that CER composition in canine skin differs from that in human skin [3]. Thus, CER formulations based on canine skin characteristics may be necessary to alleviate skin lesions in dogs.

Previous studies have shown a high proportion of CER [NS] in canine skin [5], whereas human skin contains a higher proportion of CER [NP] [7]. Furthermore, clinical investigations in dogs with AD and seborrhea have demonstrated that specific CER subclasses, particularly CER [EOP] and CER [EOS], are significantly decreased in affected skin compared with healthy controls [3,4,8]. These findings suggest that supplementation with specific CER classes, rather than those traditionally used in human-oriented formulations, may be more effective for canine skin.

In the present study, we hypothesized that targeted supplementation with CER [NS], a major component of epidermal CERs in healthy canine skin, together with CER [EOP] and CER [EOS], which show the most pronounced decreases in skin diseases, would exert beneficial effects on canine skin. To develop a canine-specific CER (cCER) and evaluate its effects on canine keratinocytes, CER mixtures were assessed for their anti-inflammatory properties, effects on keratinocyte differentiation, and CER synthesis in vitro, as well as their impact on skin barrier recovery and hair condition in dogs.

## 2. Materials and Methods

### 2.1. Culture of Canine Epidermal Keratinocyte Progenitor (CPEK) Cells

CPEK cells were obtained from CELLnTEC Advanced Cell Systems (Bern, Switzerland). The cells were maintained in Dulbecco’s Modified Eagle’s Medium (DMEM; Hyclone, Logan, UT, USA) supplemented with 10% fetal bovine serum (FBS; Hyclone) and 1% antibiotic/antimycotic solution (Hyclone) at 37 °C in a humidified incubator containing 5% CO_2_. Cells were subcultured upon reaching 80% confluency and detached using 0.25% trypsin-EDTA (Thermo Fisher Scientific, Waltham, MA, USA). For all experiments, serum-free DMEM containing 1% antibiotic/antimycotic solution was used as the experimental medium to eliminate the potential influence of serum on cellular responses. The CPEK cells were seeded into 6-well plates at a density of 4 × 10^5^ cells/well and incubated at 37 °C for 24 h. To induce cytokine expression, CPEK cells were co-cultured with *Staphylococcus pseudintermedius*, a clinical isolate obtained from a dog with a bacterial skin infection (provided by the Veterinary Medical Teaching Hospital, Konkuk University, Seoul, Republic of Korea). *S. pseudintermedius* was cultured in Tryptic Soy Broth (Difco Laboratories, Detroit, MI, USA) at 37 °C for 24 h and subsequently diluted to the appropriate density for each experiment. Synthetic CER [EOP], CER [EOS], and CER [NS] were provided by Shenzhen Dieckmann Biotechnology (Shenzhen, China). To evaluate the effects of CERs on cytokine expression, CPEK cells were exposed to CERs (10 μg/mL) and co-cultured with *S. pseudintermedius* (1 × 10^8^ CFU/mL) in Corning^®^ Transwell inserts with a 0.4 µm pore polyester membrane (Product No. 3450; Corning Inc., Corning, NY, USA) for 6 h, with dexamethasone (1 μM; Sigma-Aldrich, St. Louis, MI, USA) used as a positive control. In addition, to investigate the expression of keratin 10 (KRT10) and CER synthesis enzymes, CPEK cells were treated with CERs (10 μg/mL) for 24 h, with calcium chloride (1.2 mM, Sigma-Aldrich) or D-sphingosine (1 μM; Sigma-Aldrich) used as positive controls.

### 2.2. Quantitative Real-Time Polymerase Chain Reaction (qRT-PCR) Analysis

To analyze the mRNA expression of proinflammatory cytokines, KRT10, and CER synthesis enzymes in CPEK cells, total RNA was isolated using the RNeasy Mini Kit (Qiagen, Milan, Italy), and cDNA was synthesized using ReverTraAce™ qPCR RT Master Mix (TOYOBO, Osaka, Japan) with a C1000 Touch™ Thermal Cycler (Bio-Rad, Hercules, CA, USA). Power SYBR Green PCR Master Mix (Applied Biosystems, Warrington, UK) was used with the QuantStudio™ 1 Real-Time PCR instrument (Thermo Fisher Scientific). The thermal cycling conditions were as follows: initial denaturation at 50 °C for 2 min, followed by 95 °C for 10 min, and then 40 cycles of denaturation at 95 °C for 15 s and annealing/extension at 60 °C for 1 min. Relative gene expression levels were calculated using the 2^−ΔΔCt^ method. The primer sequences used for qRT-PCR analysis are listed in Table 1.

### 2.3. Cell Viability Assay

Canine-specific ceramide (cCER) was defined as a complex consisting of ceramide NS (CER [NS]) and ceramide EOP (CER [EOP]) at a weight ratio of 4:1 (8 µg/mL and 2 µg/mL, respectively; total 10 µg/mL), determined based on the optimization described in Section 3.2. To evaluate the cytotoxicity of cCER on CPEK cells, cell viability was assessed using the Cell Counting Kit-8 (CCK-8; Dojindo Laboratories, Kumamoto, Japan). CPEK cells were seeded into 96-well plates at a density of 1 × 10^4^ cells/well and incubated at 37 °C for 24 h. The cells were then treated with cCER at concentrations of 0.1, 1, 10, and 100 μg/mL for 24 h. Following treatment, CCK-8 solution was added to each well, and the plates were incubated for 2 h at 37 °C. Absorbance was measured at 450 nm using a microplate reader. Cell viability was expressed as a percentage relative to the untreated control group.

### 2.4. Evaluation of the Effects of the Canine-Specific Ceramide (cCER) Using Reconstructed Canine Epidermis (RCE) Models

RCE models were custom-fabricated by CleCell (Seoul, Republic of Korea) using CPEK cells based on previously established protocols [9,10]. This model forms a stratified epidermoid structure comprising a stratum corneum-like layer, stratum granulosum-like layer, and stratum spinosum-like layer. RCE models (CleCell) were pre-incubated overnight in air-liquid interface culture media (CleCell) at 37 °C in a humidified incubator containing 5% CO_2_. After stabilization, the RCE models were treated with cCER (10 μg/mL) and cultured for 72 h.

For histological analysis, canine skin and RCE models were embedded in paraffin. Paraffin-embedded sections were deparaffinized in xylene (2 × 5 min) and rehydrated through a graded ethanol series (100% ethanol 2 × 3 min, 95% ethanol 2 min), followed by rinsing in distilled water. Antigen retrieval was performed by heat-induced epitope retrieval (HIER) using citrate buffer (pH 6.0) at 95 °C for 20 min. Endogenous peroxidase activity was quenched with 3% H_2_O_2_ in methanol for 10 min. Sections were washed twice with PBS and then blocked with 5% normal serum in PBS for 1 h at room temperature. Immunohistochemistry (IHC) was performed using the following primary antibodies incubated overnight at 4 °C: anti-loricrin (LOR) antibody (Abcam, Cambridge, UK; Cat# ab176322) at 1:2000, anti-CERS3 antibody (Aviva Systems Biology, San Diego, CA, USA; Cat# ARP31648) at 1:50, and anti-ceramide (CER) antibody (Sigma-Aldrich; Cat# C8104) at 1:50. Additionally, anti-KRT10 antibody (Abcam; Cat# ab9026) was applied at a 1:200 dilution and incubated for 3 h at room temperature. After washing three times with PBS (5 min each), sections were incubated with Broad Antibody Enhancer (Polink-2 HRP Plus Broad DAB Detection System, ZSGB-BIO, Beijing, China) for 30 min, followed by Polymer-HRP reagent for 30 min at room temperature. Bound antibodies were visualized using a DAB peroxidase substrate solution, producing a black or brown color, and sections were counterstained with hematoxylin for 15–20 s. Slides were then dehydrated, cleared in xylene, and mounted. The overall staining procedure was identical for all three antibodies, with the exception of the primary antibody dilution.

In addition, RCE models were treated with Pam3CSK4 (Pam3CysSerLys4; InvivoGen, San Diego, CA, USA; Cat# tlrl-pms), a TLR2/TLR1 agonist that induces both proinflammatory and regulatory responses. The experimental groups were divided into three conditions: non-treated, Pam3CSK4-treated, and Pam3CSK4/cCER-treated. RCE models were exposed to cCER (10 μg/mL) with Pam3CSK4 treatment (10 μg/mL) for 72 h, after which samples were embedded in paraffin, sectioned, and stained with hematoxylin and eosin.

IHC staining was performed in triplicate using canine skin sections obtained from one healthy dog, and the most representative image was selected for presentation. For histological analysis, experiments were performed using two RCE models per group across three independent experiments.

### 2.5. Effects of the cCER on the Recovery of Skin Barrier Function and Hair Condition in Dogs

Changes in TEWL were evaluated using the Gpskin barrier^®^ device (G Power, Hanam, Republic of Korea) following application of a formulation containing the cCER complex (0.2% cCER in a base formulation composed of fatty alcohols, waxes, pH adjusters, purified water, and preservatives). The experimental subjects consisted of two healthy, intact male Beagle dogs, each approximately one year old. This sample size reflected practical constraints, including cost and subject availability, and the in vivo component of this study was therefore designed as an unpowered pilot evaluation. Four trials were conducted by alternating treatments on both sides of the inguinal region of each dog. To assess the effect of the cCER complex on skin barrier recovery, the skin barrier was disrupted by 20 tape-stripping procedures at each treated site. TEWL values were measured immediately after tape stripping (day 0) and subsequently on days 1, 2, 3, and 4 during daily application of the cCER complex. The rate of skin barrier recovery was calculated as follows: (%) = [100 × (TEWL on day 0 − TEWL on days 1, 2, 3, or 4)/TEWL on day 0]. To evaluate hair changes, hair shafts were collected after 4 days of cCER complex application (2.5 mg/mL). To assess changes in the cuticle surface, hair shafts were coated with gold and examined using a scanning electron microscope (SEM, SNE-4500M, SEC, Suwon, Republic of Korea). In addition, hair shafts were sectioned and stained with Nile red to analyze their lipid content. For hair keratin analysis, keratin 31 (KRT31) antibody (Abcam; Cat# ab177955) and a FITC-conjugated secondary antibody (Bethyl Laboratories, Montgomery, TX, USA; Cat# A120-101F) were used. Fluorescence-stained sections were imaged using a confocal microscope. ImageJ software (version 1.54p; NIH, Bethesda, MD, USA) was used to quantify fluorescence intensity of red and green signals corresponding to lipid content and KRT31 expression, respectively. Fluorescence intensity values were obtained from cross-sections of three hair shafts per group (non-treated and cCER-treated). All animal experiments were approved by the Institutional Animal Care and Use Committee of Jeonbuk National University prior to study initiation (NON2024-131-001, approved on 24 September 2024).

### 2.6. Statistical Analysis

**Figure 1:**
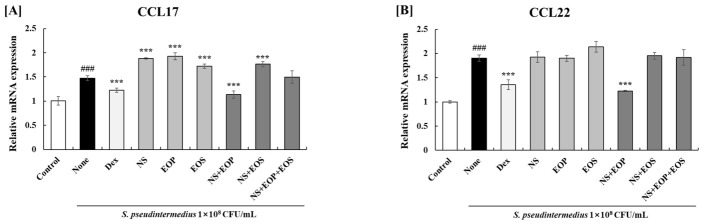
Effects of ceramides (CER) and various CER combinations (10 μg/mL) on the mRNA expression of (**A**) CCL17 and (**B**) CCL22 in CPEK co-cultured with *S. pseudintermedius*. Control, untreated CPEK without *S. pseudintermedius* co-culture; None, *S. pseudintermedius*-treated CPEK without CER treatment. TARC/CCL17, thymus activation-regulated chemokine; MDC/CCL22, macrophage-derived chemokine; Dex, dexamethasone (1 μM). All values are mean ± standard error. ^###^ *p* < 0.001 indicate a significant difference from the Control; *** *p* < 0.001 indicate a significant difference from the *S. pseudintermedius*-control.

**Figure 2:**
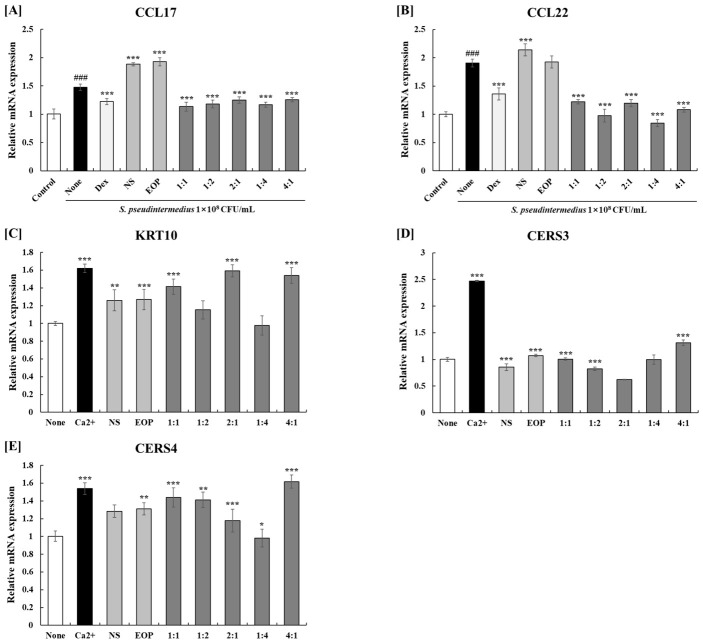
Effects of various ratios of the CER [NS]/[EOP] mixture (10 μg/mL) on the mRNA expression of (**A**) CCL17, (**B**) CCL22, (**C**) KRT10, (**D**) CERS3, and (**E**) CERS4 in CPEK co-cultured with *S. pseudintermedius*. TARC/CCL17, thymus activation-regulated chemokine; MDC/CCL22, macrophage-derived chemokine; KRT10, keratin 10; CERS3, ceramide synthase 3; CERS4, ceramide synthase 4; Dex, dexamethasone (1 μM); Ca^2+^, calcium chloride (1.2 mM). Control, untreated CPEK without *S. pseudintermedius* co-culture; None, *S. pseudintermedius*-treated CPEK without CER treatment. All values are mean ± standard error. ^###^ *p* < 0.001 indicate a significant difference from the Control; * *p* < 0.05, ** *p* < 0.01, and *** *p* < 0.001 indicate a significant difference from the *S. pseudintermedius*-control.

**Figure 3:**
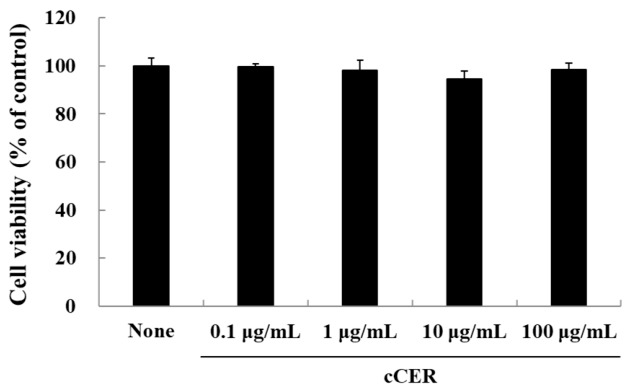
Effect of the cCER (0.1, 1, 10, and 100 μg/mL) on CPEK cell viability. All values are mean ± standard error.

**Figure 4:**
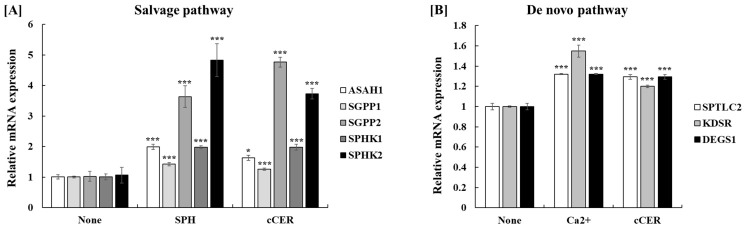
Effect of cCER complex (4:1 ratio of the mixture of CER [NS]/[EOP]) (10 μg/mL) on the mRNA expression of (**A**) ceramide salvage pathway enzymes (ASAH1, SPHK1, SPHK2, SGPP1, and SGPP2) and (**B**) de novo ceramide synthesis pathway enzymes (SPTLC2, KDSR, and DEGS1) in CPEK. ASAH1, acid ceramidase; SPHK, sphingosine kinases; SGPP, sphingosine-1-phosphate phosphatases; SPTLC2, serine palmitoyltransferase long chain base subunit 2; KDSR, 3-ketodihydrosphingosine reductase; DEGS1, dihydroceramide desaturase-1; Ca^2+^, calcium chloride (1.2 mM); SPH, sphingosine (1 μM). All values are mean ± standard error. * *p* < 0.05 and *** *p* < 0.001 indicate a significant difference from the control.

**Figure 5:**
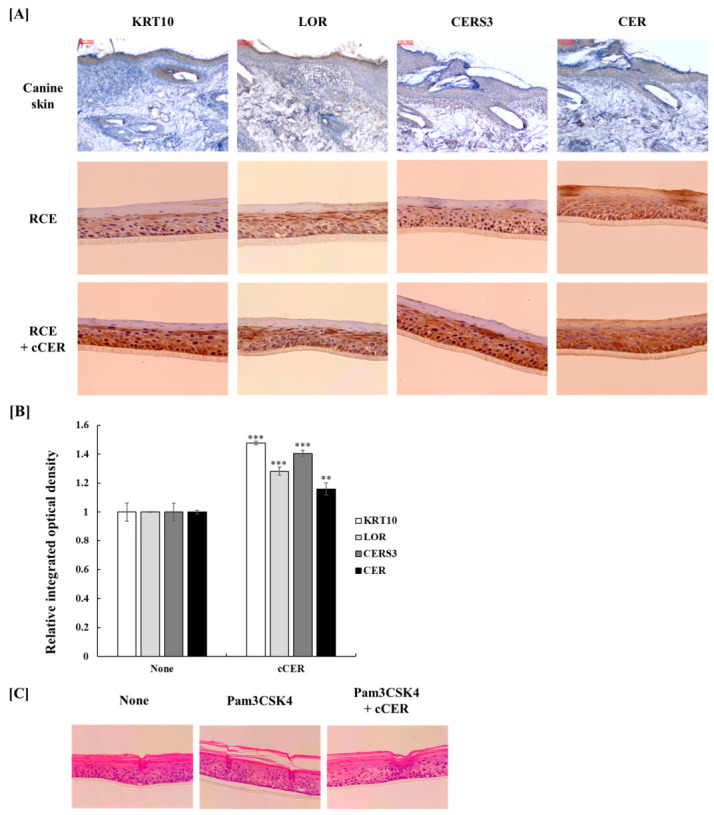
Effect of cCER (10 μg/mL) on the epidermis in RCE. (**A**) Immunohistochemical staining for KRT10, LOR, CERS3, and CER in canine skin and RCE models. (**B**) Relative integrated optical density (IOD) quantification of KRT10, LOR, CERS3, and CER expression in IHC-stained sections. (**C**) Hematoxylin and eosin staining of non-treated, Pam3CSK4-treated, and Pam3CSK4/cCER-treated RCE models. Representative images are shown. KRT10, keratin 10; LOR, loricrin; CERS3, ceramide synthase 3; CER, ceramide; RCE, reconstructed canine epidermis; Pam3CSK4, Pam3CysSerLys4 (10 μg/mL). All values are mean ± standard error. ** *p* < 0.01 and *** *p* < 0.005 indicates a significant difference from the control.

**Figure 6:**
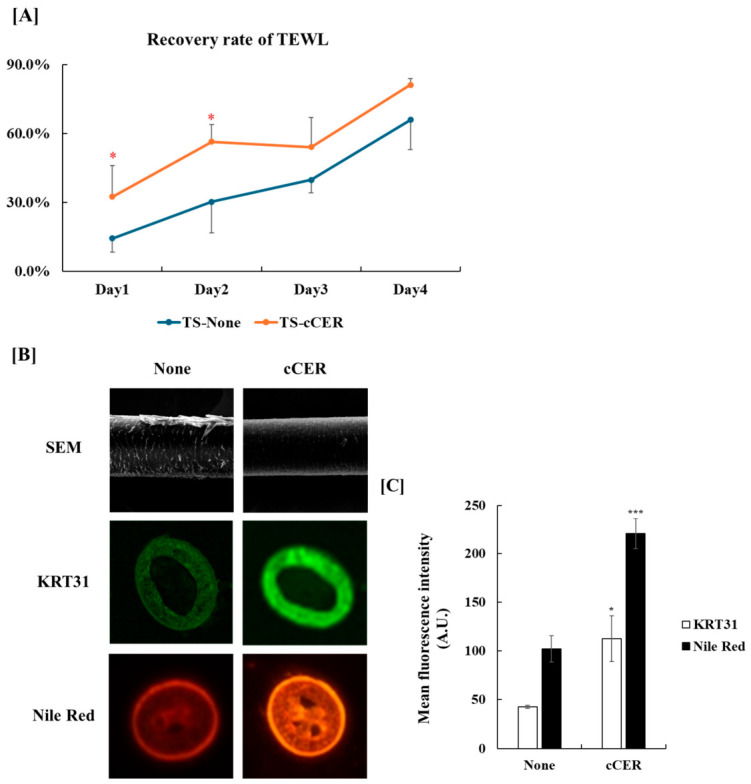
Effects of cCER-complex (0.2%) on skin and hair condition of dogs. (**A**) Recovery rate of TEWL in the skin of cCER-treated and control dogs. (**B**) Scanning electron microscopy images and Nile red and KRT31 staining of hair treated with cCER for 4 days (×600 magnification). (**C**) Quantitative image analysis of Nile red and KRT31 fluorescence intensity in cCER-treated and control hair. TS, tape stripping; TEWL, transepidermal water loss; KRT31, keratin 31. All values are mean ± standard error. * *p* < 0.05 and *** *p* < 0.005 indicate a significant difference from the control.

All experiments were repeated three times, and data are presented as the mean ± standard deviation. For multiple group comparisons (Figure 1, Figure 2, Figure 3 and Figure 4), statistical analysis was performed using one-way analysis of variance (ANOVA) followed by Dunnett’s post hoc test, using JASP software (version 0.97.1; JASP Team, University of Amsterdam, Amsterdam, The Netherlands). For two-group comparisons (Figure 5 and Figure 6), a two-tailed Student’s *t*-test was performed using Microsoft Excel. Values of *p* < 0.05 were considered statistically significant.

## 3. Results

### 3.1. Effects of CER Mixtures on Expression of Proinflammatory Cytokines

To assess the inhibitory effects of CER [NS], [EOP], and [EOS] on *S. pseudintermedius*–induced inflammation, mRNA expression levels of thymus activation–regulated chemokine (TARC/CCL17) and macrophage-derived chemokine (MDC/CCL22) were analyzed by qRT-PCR. CPEK cells co-cultured with *S. pseudintermedius* exhibited significantly increased mRNA expression of CCL17 and CCL22. Among the various combinations of CER components tested, only the mixture of CER [NS] and [EOP] significantly decreased mRNA expression of CCL17 (Figure 1A) and CCL22 (Figure 1B), to levels comparable to those observed with dexamethasone treatment (Figure 1). In contrast, other combinations of CER [NS], [EOP], and [EOS] showed no significant differences compared with the control group.

### 3.2. Optimization of the Combination Ratio: Mixture of CER [NS] and [EOP]

To determine the optimal CER [NS]+[EOP] ratio, a series of combinations was evaluated for their effects on gene expression. All tested mixtures reduced mRNA expression levels of CCL17 and CCL22 compared with the control group (Figure 2A,B). Ratios of 1:1, 2:1, and 4:1 induced upregulation of KRT10 expression (Figure 2C), whereas ratios of 1:1, 1:2, and 4:1 increased CERS3 levels (Figure 2D). Furthermore, ratios of 1:1, 1:2, 2:1, and 4:1 enhanced the expression of ceramide synthase 4 (CERS4) (Figure 2E). Notably, only the 4:1 CER [NS]+[EOP] ratio simultaneously suppressed cytokine expression and induced both KRT10 and ceramide synthase enzymes, thereby identifying this formulation as the canine-specific ceramide (cCER) used in this study (Figure 2).

### 3.3. Cytotoxicity of cCER in CPEK Cells

To confirm the safety of cCER prior to subsequent experiments, the cytotoxicity of cCER was evaluated in CPEK cells using the CCK-8 assay. Treatment with cCER at concentrations of 0.1, 1, 10, and 100 μg/mL did not significantly affect cell viability compared with the untreated control group (Figure 3), indicating that cCER exhibits no cytotoxic effects on CPEK cells at the tested concentrations.

### 3.4. Effect of the cCER on the Expression of CER Synthesis Pathway Enzymes

The impact of the cCER on CER metabolism was assessed by examining key enzymes involved in both the salvage and de novo synthesis pathways. For the salvage pathway, mRNA expression of acid ceramidase (ASAH1), sphingosine kinases (SPHK1 and SPHK2), and sphingosine-1-phosphate phosphatases (SGPP1 and SGPP2) was analyzed by RT-PCR. The de novo synthesis pathway was evaluated through analysis of mRNA expression of serine palmitoyltransferase long-chain base subunit 2 (SPTLC2), 3-ketodihydrosphingosine reductase (KDSR), and dihydroceramide desaturase-1 (DEGS1). cCER treatment significantly increased mRNA expression levels of enzymes involved in the salvage pathway, comparable to the effects observed in positive controls treated with sphingosine (Figure 4A). Moreover, enzymes associated with the de novo synthesis pathway were significantly upregulated in CPEK cells treated with cCER, similar to the levels observed in positive controls treated with calcium chloride (Figure 4B).

### 3.5. Effects of the cCER on RCE

RCE models exhibited a well-differentiated epidermis composed of multiple stratified layers. Antibody validation targeting KRT10, LOR, CERS3, and CER was performed using canine skin sections. IHC analysis demonstrated cytoplasmic staining of CERS3 and CER within the epidermal layers, while the anti-KRT10 antibody showed cytoplasmic staining in the suprabasal epidermal layers, and the anti-LOR antibody showed cytoplasmic staining in the uppermost epidermal layer. RCE models similarly exhibited cytoplasmic staining of LOR, CERS3, and CER, comparable to that observed in canine skin. In addition, RCE models supplemented with cCER showed increased expression of keratinocyte differentiation markers, including KRT10, LOR, CERS3, and CER, compared with the untreated control group (Figure 5A,B). Quantification by relative integrated optical density (IOD) analysis confirmed that RCE models supplemented with cCER showed significantly increased expression of KRT10, LOR, CERS3, and CER compared with the untreated control group (Figure 5B). Upon stimulation with Pam3CSK4, disruption of the SC and increased intercellular edema accompanied by spongiosis were observed in the epidermis (Figure 5C). In contrast, cCER treatment attenuated SC deformation, resulting in histological features comparable to those of the untreated group; notably, spongiosis was absent in the Pam3CSK4/cCER-treated group (Figure 5C).

### 3.6. Effects on the Recovery of Skin Barrier Function and Hair Conditions of Dogs

Regarding changes in TEWL, cCER-treated skin demonstrated significantly accelerated recovery rates on days 1 and 2 (Figure 6A). Furthermore, SEM analysis revealed a well-organized cuticle layer in hair treated with cCER for four days (Figure 6B). Nile red staining indicated increased lipid content in cCER-treated hair compared with untreated hair. Immunofluorescence analysis of KRT31 also showed higher signal intensity in hair treated with cCER (Figure 6B). Quantitative image analysis further demonstrated significant increases in both Nile red and KRT31 expression in hair treated with cCER (Figure 6C).

## 4. Discussion

Alterations in CER profiles, as observed in disrupted skin barrier function in canine AD, are typically characterized by disorganization of intercellular lipids within the SC [11]. Among CER classes, long-chain CERs provide greater structural stability and enhanced barrier function to intercellular lipid lamellae than short-chain CERs [12]. In the present study, the cCER complex was systematically optimized based on prior findings documenting specific CER class deficiencies in dogs with inflammatory dermatoses. Moreover, the optimized complex significantly reduced proinflammatory cytokine expression and upregulated the expression of enzymes involved in CER synthesis in cultured canine keratinocytes. In addition, application of the cCER complex increased the expression of skin barrier components in RCE models, as well as in the skin and hair of dogs.

Chemokines CCL17 and CCL22 are key mediators of Th2 cell recruitment during the acute phase of AD, and their concentrations correlate with disease severity and treatment efficacy in dogs with AD [13,14]. The present investigation demonstrated that combined administration of CER [NS] and [EOP] significantly downregulated CCL17 and CCL22, which are pivotal cytokines regulating immune responses in both the acute and chronic phases of canine AD. These findings highlight the potential of the cCER complex as a novel anti-inflammatory therapeutic strategy for managing inflammatory dermatological conditions in dogs.

Reductions in cytokine levels following CER treatment have also been reported in previous studies. Chiba et al. [15] demonstrated that CERs reduced the expression of IL-5, IL-10, and IL-13 in mast cells. In addition, CER treatments produced using genetically modified yeast significantly decreased the expression of COX-2 and TNF-α [16]. Lim et al. [17] further reported that a lipid mixture rich in CER [NP] and fatty acids of varying chain lengths resulted in significant reductions in IL-2, IL-6, IL-10, and IL-13 expression. Although the precise mechanisms underlying the anti-inflammatory effects of CERs remain incompletely understood, previous studies suggest that increased sphingosine-1-phosphate cleavage from CERs may contribute to their anti-inflammatory properties in the skin [18,19].

KRT10 and LOR, which are principal structural proteins expressed in differentiated keratinocytes, play critical roles in maintaining the mechanical stability of the epidermis [20]. The present study demonstrated that the cCER complex significantly enhanced both mRNA and protein expression of KRT10 in CPEK models and increased intracellular KRT10 and LOR protein expression in RCE models. Furthermore, treatment with the cCER complex accelerated the recovery of skin barrier function. Collectively, these findings suggest that the cCER complex reinforces the skin barrier by promoting the expression of KRT10 and LOR.

CER biosynthesis involves three major pathways: the de novo pathway, the salvage pathway, and the sphingomyelin hydrolysis pathway [21]. The de novo pathway primarily generates CERs that form the lipid barrier in the SC [22], whereas the salvage pathway recycles sphingosine into CERs that are crucial for cellular metabolism and signal transduction [23]. The sphingomyelinase pathway produces CERs through sphingomyelin hydrolysis and contributes to cell signaling, apoptosis, and stress responses [24]. Shin et al. [25] reported that topical application of CERs enhances intracellular CER production by activating the salvage pathway, which may also indirectly influence the de novo pathway. In the present study, cCER treatment increased the expression of genes associated with the salvage pathway, including ASAH1, SPHK1, SPHK2, SGPP1, and SGPP2, and elevated levels of sphingosine-1-phosphate, a key metabolite in this pathway. The coordinated activity of sphingosine-1-phosphate phosphatases (SGPP1 and SGPP2) together with ceramide synthases (CERS3 and CERS4) likely facilitated increased endogenous CER biosynthesis. Moreover, upregulation of genes involved in the de novo CER synthesis pathway, including SPTLC2, KDSR, DEGS1, CERS3, and CERS4, following cCER treatment suggests enhanced production of barrier-forming CERs through both the salvage and de novo pathways.

Each component of the cCER formulation serves distinct yet interconnected roles. CERs, including CER [NS] and CER [EOP], are crucial for maintaining skin barrier integrity and regulating inflammation, both of which are critical for skin health and disease prevention [26]. Canine AD is characterized by a significant reduction in CER levels; however, topical treatments that restore these CERs have been shown to improve clinical outcomes and support barrier recovery [27,28,29]. In this study, treatment with cCER (a 4:1 ratio of CER [NS] to CER [EOP]) promoted skin barrier formation in both in vitro and in vivo models. These findings align with previous studies indicating that applying multiple CER components in appropriate proportions provides additional benefits for skin health [30,31]. Optimal CER ratios appear particularly effective in achieving these outcomes [31]. The enhanced effects observed with the combination of CER [NS] and CER [EOP] likely arise, in part, from hydrogen bonding between their head groups, which reduces transepidermal water loss, as well as van der Waals interactions among their long hydrocarbon chains, which further reinforce barrier function [30].

This study has several limitations. First, the effects of the cCER complex were primarily assessed at the mRNA level for cytokines and CER synthesis enzymes, and further research is needed to evaluate its impact on protein expression. Second, a detailed analysis of the specific CER classes produced in response to cCER treatment has not been conducted.

Effective management of canine AD requires both anti-inflammatory activity and reinforcement of skin barrier function. The cCER complex, which exhibits anti-inflammatory effects, enhances barrier function, and promotes CER synthesis, shows preliminary promise as a topical agent for supporting skin barrier recovery. However, as the in vivo component of this study was conducted in healthy dogs as a pilot safety and recovery evaluation, we acknowledge that the current findings cannot be directly extrapolated to dogs with atopic dermatitis. Larger-scale clinical trials in dogs with AD are warranted to confirm the therapeutic efficacy and safety of the cCER complex.

## 5. Conclusions

In conclusion, this study successfully optimized a canine-specific ceramide complex consisting of CER [NS] and [EOP] at a 4:1 ratio (cCER), demonstrating its dual capacity to suppress proinflammatory cytokine expression—including CCL17 and CCL22—in *Staphylococcus pseudintermedius*-stimulated CPEK cells, while simultaneously upregulating key mediators of epidermal differentiation and ceramide biosynthesis. These findings were further supported by the RCE model and in vivo observations, which collectively confirmed that cCER enhances stratum corneum organization, accelerates skin barrier repair, and improves hair condition in dogs. Taken together, the CER [NS]/[EOP] mixture represents a promising topical therapeutic candidate to be further evaluated for the adjunctive management of canine atopic dermatitis. As the in vivo experiment was a preliminary pilot evaluation conducted in healthy dogs, further clinical studies in dogs with atopic dermatitis are warranted to validate its therapeutic efficacy and safety in larger cohorts.

## Figures and Tables

**Table 1:** Primer sequences used for qRT-PCR.

Gene ^1^	Accession No.	Primer	Sequence of Primers (5′-3′)
*GAPDH*	NM_001003142.2	Forward	AAGGCTGAGAACGGGAAACT
		Reverse	TACTCAGCACCAGCATCACC
*TARC/CCL17*	NM_001003051.1	Forward	AAGATGCTGCTCCTGGTCAC
		Reverse	GCCTGCTGATAGGAATGGCT
*MDC/CCL22*	XM_038657947.1	Forward	CTACATCCGTCACCCTCTGC
		Reverse	TCCTCATGGGTCCAGCTTC
*KRT10*	XM_038676544.1	Forward	GTCAGCAGAAACTAGCTGGGATAC
		Reverse	CTTTCCTCTTGACGCGGCTT
*CERS3*	XM_038661179.1	Forward	CCCCTTTGCTGTAACCCACA
		Reverse	ACGTGAGGCAAAGCTACTGG
*CERS4*	XM_038428816.1	Forward	CAACCAACCGCGAACTGGA
		Reverse	ACCATTCGTTGAGGCTGGAC
*ASAH1*	XM_038690109.1	Forward	GACGATGCTGGGCTGGAG
		Reverse	CTGAACCCCTGTAGGTTGGTC
*SPHK1*	XM_038675272.1	Forward	GGTTATGGACTCAGCGGGC
		Reverse	TGAGCATCAGCGTGAAGGAG
*SPHK2*	XM_038656433.1	Forward	CAGCTCTAGTCCGGGGAAGG
		Reverse	CCTGGTCTGGCCTCTGGG
*SGPP1*	XM_038673825.1	Forward	GGCCGCTGGCAGTATCCTC
		Reverse	TTAGGCAAACTAGAGAACACCAGCA
*SGPP2*	XM_038448079.1	Forward	CGGTCCTGGACGTGCT
		Reverse	CAGCAGGAAGGGCACGA
*SPTLC2*	XM_038674336.1	Forward	TGGAGGCAAGAAGGAGCTGAT
		Reverse	TCTCCTTTAAAGCCAAGGGTGGT
*KDSR*	XM_038653995.1	Forward	GTGGGCAGGATCGTCTTTGT
		Reverse	CATTGTACGGCTTCACCTCCA
*DEGS1*	XM_038671732.1	Forward	GGGCCTCTAAATCTGCTCACCT
		Reverse	GCGATCTTCCTCACCAGTGGA

^1^ *GAPDH* was used as a reference gene; *GAPDH*, glyceraldehyde-3-phosphate dehydrogenase; *TARC/CCL17*, thymus activation regulated chemokine; *MDC/CCL22*, macrophage-derived chemokine; KRT10, keratin 10; *CERS3*, ceramide synthase 3; *CERS4*, ceramide synthase 4; *ASAH1*, N-acylsphingosine amidohydrolase 1; *SPHK1*, sphingosine kinase 1; *SPHK2*, sphingosine kinase 2; *SGPP1*, sphingosine-1-phosphate phosphatase 1; *SGPP2*, sphingosine-1-phosphate phosphatase 2; *SPTLC2*, serine palmitoyltransferase long chain base subunit 2; *KDSR*, 3-ketodihydrosphingosine reductase; *DEGS1*, dihydroceramide desaturase-1.

## Data Availability

The datasets used and/or analyzed in the current study are available from the corresponding author upon reasonable request.

## Abbreviations

The following abbreviations are used in this manuscript:

CER	ceramide
CPEK	canine epidermal keratinocyte progenitor
RCE	reconstructed canine epidermis
cCER	canine-specific ceramide
TEWL	transepidermal water loss
SC	stratum corneum
AD	atopic dermatitis
Pam3CSK4	Pam3CysSerLys4
ASAH	acid ceramidase
SPHK	sphingosine kinase
SGPP	sphingosine-1-phosphate phosphatase
SPTLC2	serine palmitoyltransferase long-chain base subunit 2
KDSR	3-ketodihydrosphingosine reductase
DEGS1	dihydroceramide desaturase-1
